# Comparison of the pharmacokinetic profiles of three triterpenoids after oral administration of a cucurbitacin tablet and nanosuspension by UHPLC-MS/MS

**DOI:** 10.3389/fphar.2025.1647015

**Published:** 2025-09-10

**Authors:** Chun-nan Zhang, Zhi-bin Wang, Rui-tong Du, Shu-lu Zhang, Chu-qiao Wang, Meng Wang, Li-hong Wu, De-qiang Yang, Chen Wang

**Affiliations:** ^1^ Traditional Chinese Medicine Department, Heilongjiang Provincial Hospital, Harbin, China; ^2^ Key Laboratory of Basic and Application Research of Beiyao, Ministry of Education, Heilongjiang University of Chinese Medicine, Harbin, China; ^3^ Department of Pharmaceutical Analysis and Analytical Chemistry, College of Pharmacy, Harbin Medical University, Harbin, China

**Keywords:** cucurbitacin, nanosuspension, pharmacokinetic, rat plasma, UHPLC-MS/MS

## Abstract

**Introduction:**

Cucurbitacin, a class of triterpenoid compounds isolated from *Pedicellus Melo*, possesses various biological activities and is the primary active component of cucurbitacin tablets (CUT) used to treat chronic hepatitis and primary liver cancer. Nanosuspensions can potentially improve the oral bioavailability of pharmacopotent substances. This is the first study comparing the pharmacokinetics of three cucurbitacin triterpenoids (cucurbitacin B [CuB], cucurbitacin D [CuD], and cucurbitacin E [CuE] following oral administration of CUT and a novel *P*. *Melo* nanosuspension (MP-NPs) in rats.

**Methods:**

The plasma concentrations of these cucurbitacin triterpenoids were quantified through ultra‐performance liquid chromatography-tandem mass spectrometry (UHPLC‐MS/MS). A selective, simple, and sensitive UHPLC‐MS/MS method was developed using the positive ion mode for multiple reaction monitoring analysis. The chromatographic column used was Waters Acquity HSS T3 (1.8 μm, 2.1 × 100 mm), the column temperature was 35 °C, the flow rate was 0.3 mL/min, the injection volume was 5 μL, and the mobile phase was a gradient elution of water (A) and methanol (B). The intra‐ and inter-day precision for all analytes was <13%, and accuracy ranged from −6.41% to −4.01%

**Results:**

According to the pharmacokinetic results, when the two rat groups were orally administered with the same dose of CUT and MP-NPs, the elimination half-life (*T*_*1/2*_) of CuD and CuE was longer than that of CuB, indicating slower elimination. Compared with the CUT group, the triterpenoids in the MP-NPs group reached the maximum plasma concentration (*C*_max_) within 2 h, and both *C*_max_ and the area under the plasma concentration increased significantly.

**Discussion:**

The MP-NPs formulation significantly enhanced the oral bioavailability of cucurbitacin triterpenoids compared to conventional CUT. These findings underscore the potential of nanosuspension technology in improving the pharmacokinetic profile of cucurbitacin-based therapeutics. This study provides valuable insights for further development and clinical application of cucurbitacin nanosuspensions.

## 1 Introduction

For long, liver disorders and associated illnesses have been clinically treated with cucurbitacin tablets (CUT), a conventional over-the-counter medication (Huang et al., 2019; Chen and Zheng, 2014; Chen J et al., 2008; He et al., 1994). Triterpenoid compounds (i.e., CuB, CuD, and CuE), extracted from Cucurbitaceae plants, are the key active components of cucurbitacins. These compounds exert strong anticancer effects by controlling signaling pathways such as NF-κB, MAPK, and JAK/STAT. These effects include apoptosis induction and inhibition of tumor cell proliferation (Park et al., 2015). In particular, CuB modulates several pathways, including JAK/STAT3, Nrf2/ARE, NF-κB, AMPK, MAPK, PI3K/Akt, CIP2A/PP2A, Wnt, FAK, Notch, and Hippo-YAP, to offer robust anti-inflammatory, antioxidant, antiviral, hypoglycemic, hepatoprotective, neuroprotective, and anticancer properties (Dai et al., 2023; Garg et al., 2018; Yesilada et al., 1988; Li et al., 2023; Lin et al., 2019; Yang et al., 2020; Kim et al., 2018; Lu et al., 2012; Chen R et al., 2008). By controlling JAK/STAT3, PI3K/Akt/mTOR, and MAPK pathways, CuD prevents HepG2 cell proliferation and triggers their apoptosis (Üremiş et al., 2022; Ku et al., 2018). CuE exerts anti-inflammatory, neuroprotective, anticancer, anti-apoptotic, anti-aging, and immunomodulatory effects by regulating pathways such as PI3K/Akt, NF-κB, FAK/AKT/GSK3β, TFAP4/Wnt/β-catenin, SIRT1/Nrf2/HO-1, miR-371b-5p/TFAP4, NF-κB/NLRP3, JAK/STAT, Jak/Stat3, ERK/MAPK, and PI3K/Akt/mTOR (Wang L et al., 2023; Zheng et al., 2022; Yang et al., 2022; Brouwer et al., 2020; Attard and Martinoli, 2015). Additionally, it inhibits the proliferation of A549 human non-small-cell lung cancer cells by facilitating p62-dependent apoptosis (Üremiş et al., 2023). However, the clinical application of these compounds is limited by the poor oral bioavailability of cucurbitacins, mainly because of low water solubility and substantial first-pass effects (Hsu et al., 2023; Yu et al., 2014).

The dried peduncle of *Pedicellus Melo* fruits, a crop of great economic value in terms of cucurbitacin production, is a traditional Chinese medicinal material commonly used to treat phlegm retention, jaundice, and inflammatory diseases (Chen et al., 2024). According to modern pharmacological studies, the *P. Melo* extract is rich in CuB, CuD, and CuE, the primary active components responsible for its therapeutic effects. However, similar to traditional cucurbitacin formulations, natural extracts are poorly water-soluble and are rapidly cleared through the gastrointestinal tract, leading to insufficient systemic exposure and low bioavailability.

Nano-drug delivery systems offer novel solutions for enhancing the solubility, stability, and bioavailability of poorly soluble drugs. For example, oral *Panax notoginseng* saponin nanoparticles or nanoemulsions have significantly improved bioavailability (Lu et al., 2024). Breviscapine nanosuspensions enhance solubility and dissolution and markedly increase bioavailability in mice (Zhang et al., 2022). Polyvinylpyrrolidone K30 (PVP K30), a polymer commonly applied in nano-drug delivery systems, has been successfully used for multiple traditional Chinese medicine components (Waleka et al., 2021). For instance, PVP K30 inhibits the crystallization of artemisinin to improve its transdermal permeability (Xue et al., 2021); reduces paclitaxel crystallinity, thereby improving its solubility (Shikov et al., 2009); and increases the stability and bioavailability of curcumin (Chen et al., 2022).

Nanotechnology is thus promising in overcoming the bottleneck of low absorption efficiency in traditional herbal extracts. CuB, CuD, and CuE are poorly water-soluble, which limits their clinical efficacy. To date, these compounds are predominantly administered as oral tablets, but adequate absorption of their active ingredients remains difficult. Therefore, water-soluble formulations must be developed to improve their effectiveness and ease of use. Preparing nanodispersions is effective for improving drug dispersion and solubility, thereby elevating bioavailability. Solid dispersion can significantly improve the solubility and dissolution rate of many active ingredients in drugs, which are otherwise poorly water-soluble (Jacob et al., 2020; Wei et al., 2022; Fathi-Karkan et al., 2024; Wang L et al., 2017). To optimize the administration method, the *P. Melo* extract was prepared, and MP-NPs were formulated using PVP K30 as a material. Subsequently, the pharmacokinetic differences between CUT and MP-NPs were compared. Then, three high-content triterpenoid compounds, namely, CuB, CuD, and CuE, were detected from the extract, and pharmacokinetic studies were conducted on different formulations. This study is the first to quantify pharmacokinetic differences among CuB, CuD, and CuE in two formulations, offering valuable information about the drug’s absorption and distribution in the body. A deeper understanding of the drug’s pharmacokinetics will aid in optimizing therapeutic strategies.

**FIGURE 1:**
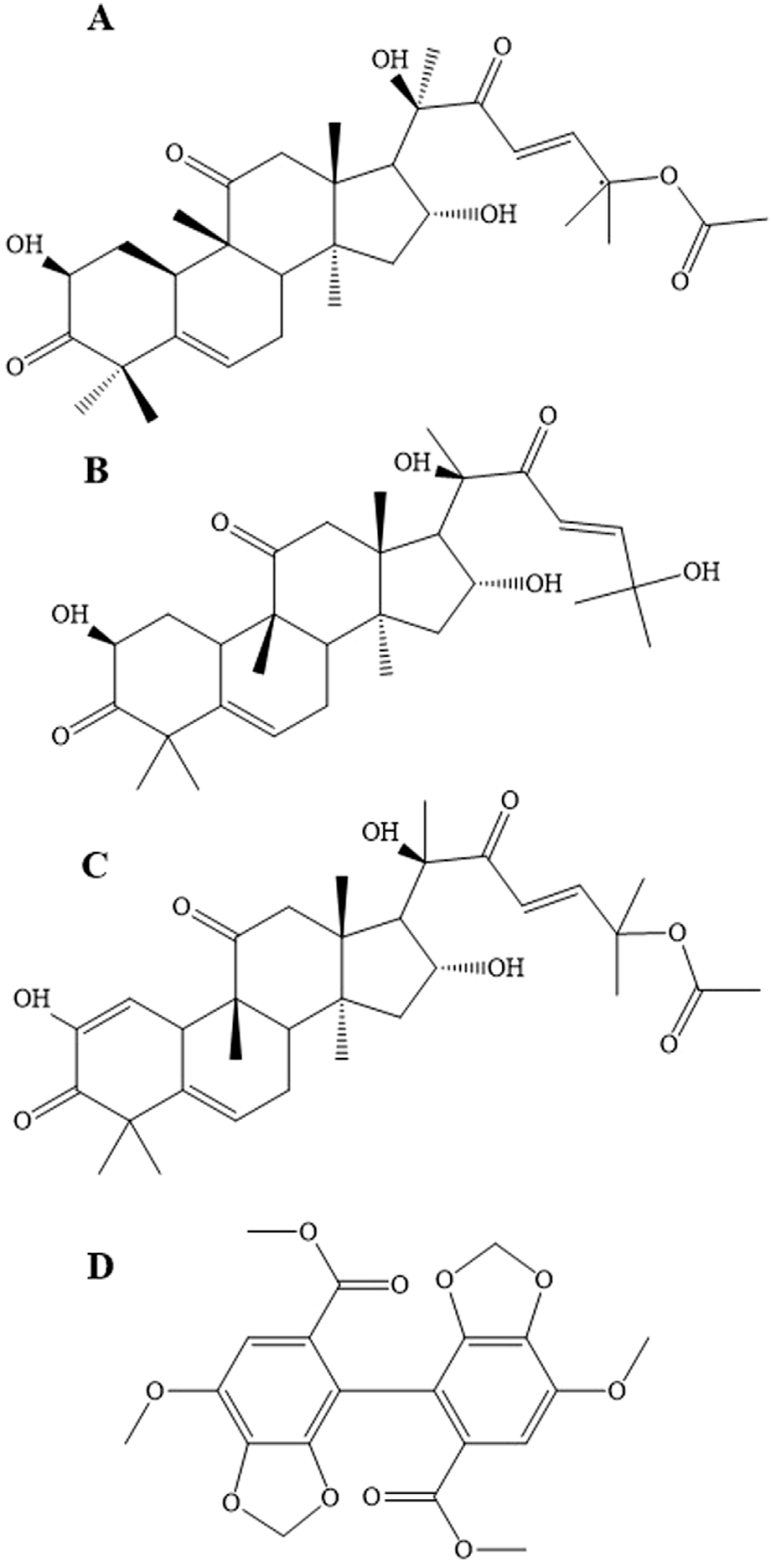
The chemical structures of analytes. **(A)** Cucurbitacin B; **(B)**Cucurbitacin D; **(C)** Cucurbitacin E; **(D)** IS.

The study aims to provide an accurate and effective UHPLC-MS/MS approach for the concurrent examination of active components from the CUT and MP-NPs groups, including CuB, CuD, and CuE, with bifendate employed as the internal standard (IS) (Figure 1). Pharmacokinetic analysis was also performed for the first time after a single oral administration of CUT and MP-NPs in rats, offering valuable insights to improve the oral availability of insoluble drugs during clinical application.

## 2 Materials and methods

### 2.1 Materials

The reference substances of cucurbitacin B (CuB; lot: Z02M7X10137; purity ≥98%), cucurbitacin D (CuD; lot: DSTDH008101; purity ≥98%), and cucurbitacin E (CuE; lot: Z01M7S10309; purity ≥98%) were purchased from Shanghai Yuanye Biotechnology Co., Ltd. Bifendate (lot: 73536-69-3; purity ≥98%) was purchased from Chengdu Must Bio-Technology (Chengdu, Sichuan Province, China) as an IS. All other chemical reagents, including methanol and formic acid (Dikma Technologies, Beijing, China), were of HPLC quality. *P. Melo* (RP20240313) was purchased from Shaanxi Pioneer Biotech Co., Ltd. And identified by Professor Zhenyue Wang of Heilongjiang University of Chinese Medicine.

### 2.2 Instruments and UHPLC-MS/MS conditions

**FIGURE 2:**
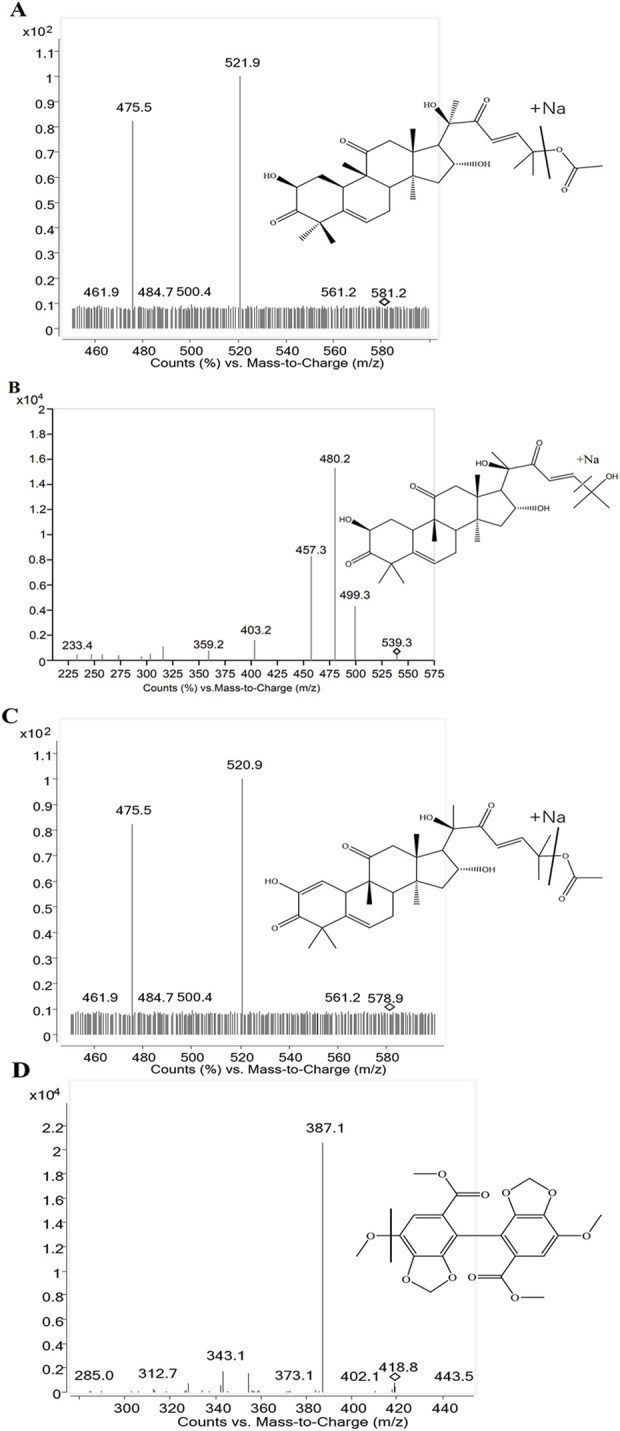
Product ion mass spectra of the analytes. **(A)** Cucurbitacin B, **(B)** Cucurbitacin D, **(C)** Cucurbitacin E, **(D)** IS.

An Agilent 6430 triple quadrupole mass spectrometer with an ESI source interface (Agilent Technologies, Santa Clara, CA, United States) was used in conjunction with an Agilent 1290 UHPLC system (Agilent Technologies). A Waters ACQUITY HSS T3 Column (1.8 μm, 2.1 × 100 mm) (Waters, Manchester, UK) was also used for the separations. Gradient elution was performed using water (A) and methanol (B) as mobile phases. The gradient elution procedure was used for 0.0–1.0 min, 80%–80% B; 1.0–1.5 min, 80%–90% B; 1.5–3.0 min, 90%–90% B; 3.0–3.2 min, 90%–80% B; and 3.2–4 min, 80%–80% B. The analytical temperature was 35 °C, and the flow rate was 0.3 mL/min. An injection of 5 μL volume was administered, after which the needle was washed. Positive ion-mode analysis was performed for the samples. The capillary voltage was set to 4500 V, source temperature to 100 °C, and the desolvation temperature to 350 °C (Wang et al., 2014). High-purity nitrogen was used as the nebulizing gas, and nitrogen was used as the drying gas at a flow rate of 11 L/min. The Agilent Mass Hunter workstation was used to gather the experimental data (Wang Z et al., 2023). Using specific precursor and product ion transitions at *m/z* 581.2→521.9 for CuB, *m/z* 539.3→480.2 for CuD, *m/z* 578.9→520.9 for CuE, and *m/z* 418.9→387.1 for IS at various fragmentor voltage (FV) and collision energy (CE) conditions listed in Table 1, quantification was accomplished using multiple reaction monitoring (MRM) mode. The production mass spectra of the three analytes and IS are displayed in Figure 2.

**TABLE 1:** MRM parameters of the three analytes and IS.

Compounds	Transition	Fragmentor (V)	Collision energy (eV)	Quantifier ions	Polarity
IS	418.9→387.1	120	7	343.1	Positive
Cucurbitacin B	581.2→521.9	122	30	475.5	Positive
Cucurbitacin D	539.3→480.2	100	17	499.3	Positive
Cucurbitacin E	579.4→520.9	100	40	434.6	Positive

### 2.3 Preparation of standards and QC samples

CuB (4.953 mg), CuD (4.603 mg), and CuE (4.544 mg) were accurately weighed and converted to 10 mL in methanol to prepare a single mother liquor of 0.4953, 0.4603, and 0.4544 mg/mL, respectively. The IS 5.504 mg was dissolved in methanol in a 20 mL volumetric flask, and a 0.275 mg/mL stock solution was obtained. The calibration solution was prepared by using the mother liquor gradient dilution method.

Following the necessary addition of blank plasma and standard solutions to the samples for the standard calibration curves, the final concentrations obtained were 2.109, 4.212, 10.50, 21.04, 42.10, 210.4, and 420.3 ng/mL for CuB; 10.08, 20.21, 50.52, 101.2, 202.7, 404.2, and 808.2 ng/mL for CuD; 2.103, 4.221, 10.54, 21.13, 42.09, 210.3, and 420.1 ng/mL for CuE. These concentrations were achieved by spiking 100 μL of blank plasma with 10 μL of IS and 100 μL of a mixed standard solution containing the three compounds. Drug-free plasma was used to create QC samples at four different concentration levels, HQC are 300.2, 600.3, and 300.8 ng/mL; MQC are 150.1, 300.1, and 150.4 ng/mL; LQC are 5.102, 25.31, and 4.912 ng/mL; LLOQ are 2.109, 10.08, and 2.103 ng/mL for CuB, CuD, and CuE. All working solutions were stored at 4 °C throughout the experiment.

### 2.4 *Pedicellus Melo* nanosuspension preparation

A specific quantity of *P. Melo* was homogenized, and 8-fold volumes of methanol were added to the sample. The mixture was then subjected to three sequential ultrasonic extractions (for 40 min each) at room temperature. Following each extraction cycle, the slurry was vigorously shaken, filtered through a 0.22 μm membrane filter, and the filtrate was collected. The pooled filtrates were concentrated via rotary evaporation under reduced pressure to obtain the crude *P. Melo* extract. Simultaneously, a 20% (w/v) PVP K30 solution was prepared by dissolving the polymer in deionized water. The solution was magnetically stirred (600 rpm) at 60 °C in a water bath until a homogeneous, clear aqueous phase was achieved.

### 2.5 Animal experiments

A total of 12 male Sprague Dawley (SD) rats (weight: 220 ± 20 g) were provided by the Animal Experiment Center of Heilongjiang University of Traditional Chinese Medicine (Production license number: SYXK (Hei) 2021-010). The Laboratory Animal Ethics Committee of Heilongjiang University of Traditional Chinese Medicine approved the study (Approval No.: 2024053114).

Animals were randomly assigned to the following two experimental groups (*n* = 6 per group): CUT and MP-NPs. Treatments were administered once daily for seven consecutive days, with the plasma sampling performed at predetermined time points after administration. Dosage was calculated based on human-equivalent doses converted to rat pharmacokinetic parameters using interspecies scaling factors. The human-rat dose conversion procedure was used to determine the dose of CUT per rat to be 0.03 g. The recommended daily dosage for humans is 6 tablets. The dose of MP-NPs per rat was determined to be 0.06 g per rat using the human-rat dose conversion formula, whereas the dose of *P. Melo* for human usage was 3 g, with reference to the Pharmacopoeia of the People’s Republic of China 2025.

At 0.083, 0.25, 0.50, 1, 1.5, 2, 2.5, 3, 4, 6, 8, 12, and 24 h following oral administration of CUT and MP-NPs, 0.25 mL of plasma samples were sampled by rapidly collecting and centrifuging at 4 °C, followed by freezing at −20 °C for further analysis (Yu et al., 2023).

### 2.6 Plasma samples preparation

Blank plasma sample (100 μL) was vortexed for 60 s after adding 100 μL of IS. To guarantee complete extraction, 3 mL of dichloromethane was then added to each sample, followed by vortexing for 120 s. Subsequently, the samples were centrifuged for 10 min at 4 °C and 3,500 rpm. At 40 °C, the top organic phase was carefully moved into a sterile tube, dried off with a mild stream of nitrogen gas, and then reconstituted in 100 μL of the original mobile phase. Following a 120 s vortex, the extracts were filtered through a 0.22 μm Millipore filter before subjecting them to quantitative analyses by injecting 5 μL of them into the UHPLC-MS/MS apparatus.

## 3 Results

### 3.1 Methods validation

#### 3.1.1 Selectivity

**FIGURE 3:**
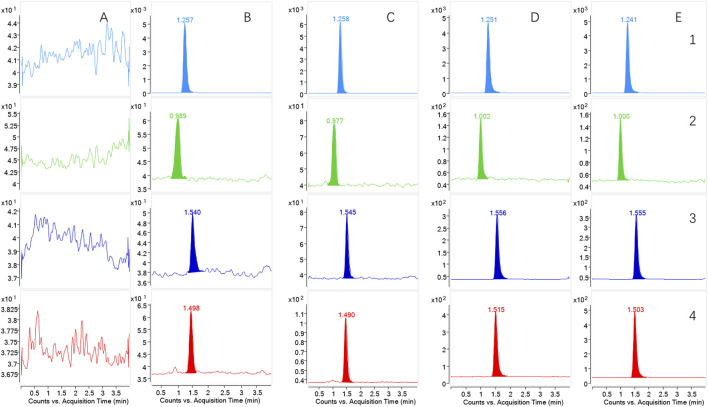
The chromatograms of the analytes in rat plasma. (1) IS; (2) Cucurbitacin D; (3) Cucurbitacin B; (4) Cucurbitacin E. **(A)** blank plasma; **(B)** plasma samples spiked with the analytes and IS at LLOQ; **(C)** blank plasma with the analytes and IS at MQC; **(D)** the plasma samples obtained at 2.5 h following the oral administration of CUT group (*n* = 6); **(E)** the plasma samples obtained at 2 h following the oral administration of MP-NPs (PVP-K30) group (*n* = 6).

Under the UHPLC-MS/MS conditions, the analyte was effectively separated. The IS, CuB, CuD, and CuE exhibited retention times of 1.258, 0.9890, 1.545, and 1.490 min, respectively. Figure 3 presents representative MRM chromatograms: (A) blank plasma; (B) plasma samples spiked with the analytes and IS at LLOQ; (C) blank plasma with the analytes and IS at MQC; (D) plasma samples obtained at 2.5 h after CUT was administered orally; and (E) plasma samples obtained at 2 h after MP-NPs were administered orally. These results confirmed that endogenous substances did not interfere with the analytes and IS during analysis.

#### 3.1.2 Linearity and sensitivity

Table 2 presents the linear regression equations for quantification. All *R* ^*2*^ exceeded 0.9914, demonstrating robust linearity across the validated concentration ranges. LLOQs for CuB, CuD, and CuE were 2.109, 10.08, and 2.103 ng/mL. These values fulfilled the sensitivity requirements for pharmacokinetic analysis.

**TABLE 2:** The calibration curves, linear ranges and LLOQs of three analytes.

Compounds	Regression equation	*R* ^*2*^	Linear ranges (ng/mL)	LLOQ (ng/mL)
Cucurbitacin B	*Y* = 4.951*10^−3^ *X* - 3.680*10^−2^	0.9914	2.109–420.0	2.109
Cucurbitacin D	*Y* = 5.792*10^−5^ *X* + 1.240*10^−2^	0.9940	10.08–808.0	10.08
Cucurbitacin E	*Y* = 2.761*10^−3^ *X* - 2.023*10^−2^	0.9940	2.103–420.0	2.103

#### 3.1.3 Precision and accuracy

Precision and accuracy were evaluated by analyzing QC samples at four concentrations: LLOQ, LQC, MQC, and HQC. Intra- and inter-day precision were assessed over three consecutive days. For all three analytes (CuB, CuD, and CuE), the precision values remained below 13% for both intra- and inter-day measurements, meeting the acceptance criteria for validation using the bioanalytical method. Accuracy, expressed as RE%, ranged from −3.987% to 9.047% across all QC levels (Table 3). Negative RE values indicated slight underestimation, whereas positive values reflected overestimation relative to nominal concentrations. These results unveiled that the analytical procedure was reliable and accurate for quantifying cucurbitacins in biological matrices.

**TABLE 3:** The intra-day and inter-day precisions and accuracies of the three analytes (*n* = 6).

Compounds	Spiked concentration (ng/mL)	Measured concentratio (ng/mL)	Intra-day		Inter-day	
			Accuracy (RE %)	Precision (RSD %)	Accuracy (RE %)	Precision (RSD %)
CuB	2.109	2.213 ± 0.2721	−2.243	9.053	8.373	12.43
	5.272	5.202 ± 0.4821	1.722	8.042	2.026	9.193
	150.1	147.3 ± 6.861	−1.054	3.974	−1.882	4.662
	300.2	288.2 ± 16.12	−2.453	4.074	−3.987	5.592
CuD	10.10	9.942 ± 1.114	−6.131	12.97	−1.803	11.19
	25.25	24.90 ± 2.060	−5.074	7.376	−1.584	8.264
	300.1	295.8 ± 9.022	−0.802	3.364	−1.422	3.051
	600.2	590.9 ± 16.80	−1.833	2.332	−1.554	2.845
CuE	2.103	2.082 ± 0.2021	−1.542	6.590	5.895	9.648
	5.257	5.341 ± 0.4913	6.701	10.41	9.047	9.112
	150.4	147.7 ± 6.652	−1.373	1.914	−1.775	4.501
	300.8	289.1 ± 21.71	−5.322	9.075	−3.903	7.518

#### 3.1.4 Extraction recovery and matrix effect

Table 4 presents the extraction recoveries of the three analytes across three QC levels, which ranged from 86.56% to 93.69%, 79.23%–83.20%, and 83.50%–91.87%, respectively. The matrix effects for the three analytes ranged from 97.02% to 106.3%. These results revealed negligible matrix effects on the analytes, indicating the appropriateness of the extraction solvent used.

**TABLE 4:** Matrix effect and extraction recovery for the three analytes (*n* = 6).

Compounds	Spiked concentration (ng/mL)	Extraction recovery		Matrix effect	
		Mean (%)	RSD (%)	Mean (%)	RSD (%)
CuB	5.272	86.56	7.512	99.65	11.98
	150.1	89.79	6.023	106.3	4.553
	300.2	93.69	6.425	100.3	9.053
CuD	25.30	79.23	5.997	99.72	11.60
	300.1	82.78	6.542	97.80	9.674
	600.2	83.20	10.52	103.3	6.522
CuE	5.257	83.50	10.37	99.20	7.505
	150.4	87.93	10.19	101.4	5.142
	300.8	91.87	7.881	97.02	9.794

#### 3.1.5 Stability

Table 5 outlines the stability of LQC, MQC, and HQC in the rat plasma under different storage conditions. CuB, CuD, and CuE remained stable under the following conditions: storage at −20 °C, ambient temperature (25 °C) for 4 h, three freeze-thaw cycles (−20 °C/room temperature) over 2 weeks, and 12 h storage at 4 °C post-sample preparation. The RSD for these conditions was ≤11.01%, confirming minimal variability. Thus, the analytes were not significantly degraded throughout all stages of sample handling and analysis.

**TABLE 5:** The stability of the three analytes under different storage conditions (*n* = 6).

Compounds	Spiked concentration	Stability (RE%)			
	(ng/mL)	Post-preparative	Short-term	Long-term	Freeze-thaw
CuB	5.272	−6.702	−6.465	4.471	−3.215
	150.1	−10.45	−3.932	−11.01	−7.542
	300.2	−8.081	−6.496	−8.414	−6.654
CuD	25.30	−3.854	4.262	5.336	−3.242
	300.1	−8.805	−4.154	−5.533	−8.420
	600.2	7.032	−5.861	−2.272	−7.765
CuE	5.257	6.711	6.050	8.213	7.947
	150.4	−2.053	−4.192	5.814	1.424
	300.8	−8.572	−3.143	−3.035	−1.361

### 3.2 Pharmacokinetic studies

**FIGURE 4:**
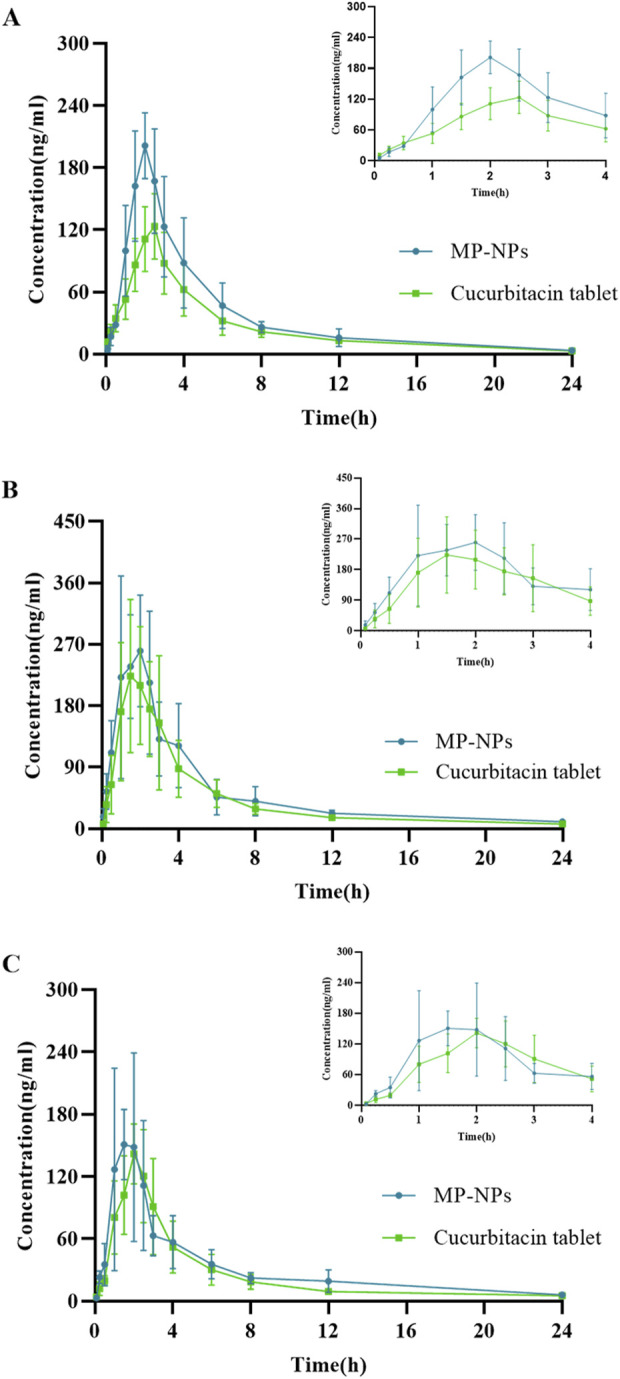
Mean concentration-time profiles of the analytes in rat plasma after oral administration of CUT and MP-NPs(PVP-K30) (*n* = 6, mean ± SD). **(A)** Cucurbitacin B; **(B)** Cucurbitacin D; **(C)** Cucurbitacin E.

The established UHPLC-MS/MS method was applied to simultaneously measure the plasma concentrations of the three analytes in rats following dose administration. Pharmacokinetic parameters have attracted attention in exploring drug absorption and distribution. These parameters include *T*_1/2_, *C*_max_, *T*_max_, and *AUC* (Table 6). The average plasma concentration-time curve is presented in Figure 4. The pharmacokinetics of SD rats following oral administration of CUT and MP-NPs were investigated through UHPLC-MS/MS. The plasma concentrations of the three analytes were calculated. The *T*_max_ values of these analytes gradually increased. The MP-NPs group reached *C*_max_ more rapidly than the CUT group. *AUC*_0→t_ and $AUC_{0\to \infty }$ also demonstrated that MP-NPs were superior to CUT preparations, suggesting that MP-NPs had higher *AUC in vivo*, possibly due to the increased drug bioavailability and absorption by the nanoparticles, thereby enhancing drug accumulation in the body.

**TABLE 6:** Pharmacokinetic parameters of the three compounds in the CUT group and the MP-NPs group rat plasma after oral administration of cucurbitacin tablet and MP-NPs(PVP-K30) (*n* = 6, mean ± SD). (Note: * for p < 0.05, and ** for p < 0.01).

Compounds	Group	*C*_max_ (ng/mL)	*T*_max_ (h)	*T*_1/2_ (h)	*AUC*_0→t_ (ng·h/mL)	$AUC_{0\to \infty }$ (ng·h/mL)
CuB	CUT	142.1 ± 22.24	2.421 ± 0.3810	4.871 ± 1.121	617.9 ± 88.56	708.1 ± 77.29
	MP-NPs	228.6 ± 11.97**	1.922 ± 0.3831*	4.569 ± 1.033	875.5 ± 217.1*	975.7 ± 255.4*
CuD	CUT	338.8 ± 24.37	1.832 ± 0.6834	5.328 ± 0.9912	1,033 ± 167.2	1,157 ± 167.8
	MP-NPs	364.7 ± 69.88*	1.752 ± 0.6102	6.967 ± 2.923	1,259 ± 260.9*	1,481 ± 225.8*
CuE	CUT	166.2 ± 13.84	2.253 ± 0.5206	5.991 ± 1.230	602.9 ± 125.4	682.3 ± 126.3
	MP-NPs	236.3 ± 31.87**	1.674 ± 0.6121*	7.053 ± 2.701	751.6 ± 149.6*	972.6 ± 268.7*

## 4 Discussion

### 4.1 Optimization of UHPLC-MS/MS conditions

Mass spectrometric conditions, including ionization and parameters, were first optimized. A higher response was observed in the ESI positive-ion mode than in the negative-ion mode. Mass spectral parameters of the compounds varied based on the instrument and operating conditions (Abdollahi et al., 2024; Bali et al., 2011; Aluri et al., 2023). The MRM mode was employed for testing the precursor and product ions of the three compounds and IS to ensure reliability. Optimal ion transition pairs for quantitation were identified: *m/z* 581.2→ 521.9 for CuB, *m/z* 539.3→ 480.2 for CuD, *m/z* 578.9→ 520.9 for CuE, and *m/z* 418.9→ 387.1 for IS in the MRM mode. The effects of different mobile phase combinations (acetonitrile–water and methanol–water) and buffers (ammonium formate, formic acid, ammonium acetate, and acetic acid) on chromatographic behavior were examined. The mobile phase selected was methanol–water, with a column temperature and flow rate of 35 °C and 0.3 mL/min, respectively. The injection volume was 5 μL, and the total run time was 4 min.

### 4.2 Selection of IS

An appropriate IS with good stability that remains unchanged during sample processing and analysis must be selected for biological sample analysis. An unstable IS may lead to errors in the quantitative analysis. This study explored the effects of m-nitrophenol and bifendate as potential IS. When bifendate was detected in the positive-ion mode, it had a retention time with a significant separation from the analyte, generating symmetrical and sharp peaks, which indicated its stability and sensitivity. Therefore, the bifendate was selected as the IS in the experiment.

### 4.3 Pharmacokinetic studies

Cucurbitacin I (CuI) is a tetracyclic triterpenoid compound primarily found in Cucurbitaceae plants. CuB, CuD, CuE, and CuI all had cucurbitane-type triterpenoid parent nuclei, but exhibited varying substituents, affecting solubility and bioavailability. CuI is well-known for exerting powerful anti-cancer properties, particularly by regulating the JAK/STAT and PI3K/Akt pathways (Xu et al., 2022; Üremiş et al., 2022; Zhu et al., 2018). CuB, CuD, and CuE also exert equally strong anti-cancer, hepatoprotective, and anti-inflammatory effects compared with CuI. In *P. Melo*, the content of CuB, CuD, and CuE was significantly higher than that of CuI, and pharmacokinetic data on CuB, CuD, and CuE (especially components in TCM nanosuspensions) in *P. Melo* remain scarce. Our MP-NP formulations specifically address the bioavailability limitations of these understudied cucurbitacins. Therefore, this study primarily investigated the pharmacokinetics of CuB, CuD, and CuE nanosuspensions. The pharmacokinetic studies revealed that MP-NPs were substantially advantageous over CUT in terms of the drug absorption rate, *C*_max_, AUC, and *T*_1/2_, possibly because of the nanosuspension characteristics of MP-NPs (such as larger surface area, higher solubility, and slower drug release). These characteristics can improve drug bioavailability and prolong its action time in the body. For example, the MP-NPs group reached *C*_max_ more quickly after absorption than the CUT group (*P* < 0.05), potentially because of the nanosuspension characteristics of MP-NPs. MP-NPs may have better bioavailability or higher solubility, and so, the drug is more easily absorbed into the bloodstream. For CuD and CuE, the *T*_1/2_ of MP-NPs was generally longer than that of CUT. For instance, the *T*_1/2_ of CuD was 6.967 h (MP-NPs) *versus* 5.328 h (CUT), and for CuE, it was 7.053 h (MP-NPs) *versus* 5.991 h (CUT). This suggests that nanosuspension formulations have a longer retention time in the body, likely due to their physicochemical properties, which allow for slow release. AUC values of the three compounds were significantly higher in the MP-NPs group (*P* < 0.05). *AUC*_0→t_ for CuB was 875.5 ng·h/mL (MP-NPs) *versus* 617.9 ng·h/mL (CUT). It was 1,259 ng·h/mL (MP-NPs) *versus* 1,033 ng·h/mL (CUT) for CuD and 751.6 ng·h/mL (MP-NPs) *versus* 602.9 ng·h/mL (CUT) for CuE (Abdollahi et al., 2024; Bali et al., 2011). This suggests that MP-NPs have a higher *AUC*, likely because their nanosuspension enhances the drug’s bioavailability and absorption rate in the body, thereby increasing drug accumulation.

Because of the polymeric nature of PVP K30, a high-molecular-weight stabilizer used for MP-NPs preparation, MP-NPs significantly improved triterpenoid solubility, likely due to reduced drug particle size and the increased specific surface area (Abdollahi et al., 2024; Bali et al., 2011; Aluri et al., 2023; Wang Z et al., 2017; Sun et al., 2024; Zhang, 2018; Roos et al., 2017; Wang et al., 2020). PVP K30 stabilizes the nanocrystal system primarily through steric hindrance. Its long polymer chains adsorb onto the nanoparticle surfaces, forming a physical barrier preventing particle aggregation through steric repulsion. This spatial barrier effectively counters attractive forces (e.g., van der Waals forces) between nanocrystals, thereby preserving their reduced size and preventing agglomeration, which is crucial for maintaining the high specific surface area and the resultant increased solubility. It is also crucial for improving its dissolution rate and promoting absorption. This experiment offers a valuable reference for the exploration of various dosage forms. The toxicology and pharmacodynamics of this preparation will be explored to continue to improve nanosuspension-related research data.

## 5 Conclusion

We here developed and validated a precise, stable, and sensitive UHPLC-MS/MS method with excellent extraction recovery, matrix effects, precision, and accuracy. This method was used to simultaneously measure CuB, CuD, and CuE content in CUT and MP-NPs following their oral administration, followed by that in rat plasma. As a nanosuspension dispersion system, MP-NPs significantly improved the oral bioavailability of the three triterpenoids compared with CUT, thereby increasing the plasma concentration of the analytes. The study results provide a foundation for clinical studies investigating the release rate, absorption, and metabolism of different dosage forms.

## Data Availability

The original contributions presented in the study are included in the article/supplementary material, further inquiries can be directed to the corresponding author.
